# (*E*)-4-[(1,5-Dimethyl-3-oxo-2-phenyl-2,3-dihydro-1*H*-pyrazol-4-yl)imino­meth­yl]-2-meth­oxy­phenyl 4-bromo­benzene­sulfonate

**DOI:** 10.1107/S1600536812010057

**Published:** 2012-03-10

**Authors:** Zhong-Yu Duan, Guo-Li Ma, Li-Ping Yang

**Affiliations:** a College of Chemical Engineering, Hebei University of Technology, Tianjin 300130, People’s Republic of China

## Abstract

In the title compound, C_25_H_22_BrN_3_O_5_S, the central benzene ring makes dihedral angles of 4.41 (10), 67.09 (9) and 62.05 (10)°, respectively, with the pyrazolone, bromo­benzene and terminal phenyl rings. The dihedral angle between the pyrazolone and phenyl rings is 57.75 (11)°. In the crystal, two pairs of C—H⋯O hydrogen bonds link the mol­ecules into inversion dimers. A weak intra­molecular C—H⋯O hydrogen bonds is also observed.

## Related literature

For general background to the use of Schiff base derivatives in the development protein and enzyme mimics, see: Santos *et al.* (2001 ▶). For closely related crystal structures, see: Guo *et al.* (2010 ▶); Han *et al.* (2008 ▶). For reference bond-length data, see: Allen *et al.* (1987 ▶).
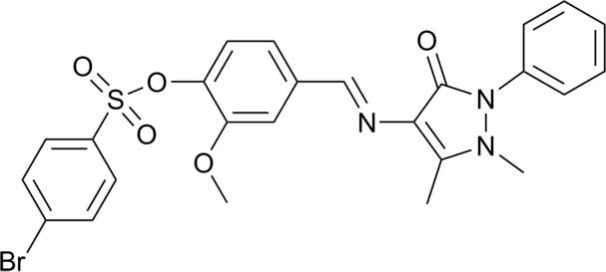


## Experimental

### 

#### Crystal data

C_25_H_22_BrN_3_O_5_S
*M* *_r_* = 556.43
Triclinic, 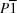

*a* = 7.271 (2) Å
*b* = 12.654 (4) Å
*c* = 13.645 (5) Å
α = 88.252 (15)°
β = 85.695 (14)°
γ = 73.623 (12)°
*V* = 1201.0 (7) Å^3^
*Z* = 2
Mo *K*α radiation
μ = 1.84 mm^−1^
*T* = 294 K
0.25 × 0.20 × 0.13 mm

#### Data collection

Bruker SMART APEX CCD area-detector diffractometer
Absorption correction: multi-scan (*SADABS*; Sheldrick, 1996 ▶) *T*_min_ = 0.628, *T*_max_ = 0.787
10073 measured reflections
4225 independent reflections
3239 reflections with *I* > 2σ(*I*)
*R*_int_ = 0.034

#### Refinement

*R*[*F* ^2^ > 2σ(*F* ^2^)] = 0.025
*wR*(*F* ^2^) = 0.059
*S* = 0.95
4225 reflections
319 parameters
H-atom parameters constrained
Δρ_max_ = 0.26 e Å^−3^
Δρ_min_ = −0.40 e Å^−3^

### 

Data collection: *SMART* (Bruker, 1999 ▶); cell refinement: *SAINT* (Bruker, 1999 ▶); data reduction: *SAINT*; program(s) used to solve structure: *SHELXS97* (Sheldrick, 2008 ▶); program(s) used to refine structure: *SHELXL97* (Sheldrick, 2008 ▶); molecular graphics: *SHELXTL* (Sheldrick, 2008 ▶); software used to prepare material for publication: *SHELXTL*.

## Supplementary Material

Crystal structure: contains datablock(s) I, global. DOI: 10.1107/S1600536812010057/is5089sup1.cif

Structure factors: contains datablock(s) I. DOI: 10.1107/S1600536812010057/is5089Isup2.hkl

Supplementary material file. DOI: 10.1107/S1600536812010057/is5089Isup3.cml

Additional supplementary materials:  crystallographic information; 3D view; checkCIF report

## Figures and Tables

**Table 1:** Hydrogen-bond geometry (Å, °)

*D*—H⋯*A*	*D*—H	H⋯*A*	*D*⋯*A*	*D*—H⋯*A*
C13—H13⋯O5	0.93	2.32	3.020 (3)	132
C22—H22⋯O1^i^	0.93	2.51	3.394 (3)	158
C12—H12⋯O5^i^	0.93	2.52	3.212 (3)	131

Symmetry code: (i) 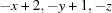.

**Fig. 1.**
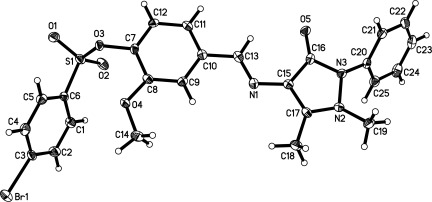
The molecular structure of the title compound, with displacement ellipsoids for non-H atoms drawn at the 50% probability level.

**Fig. 2.**
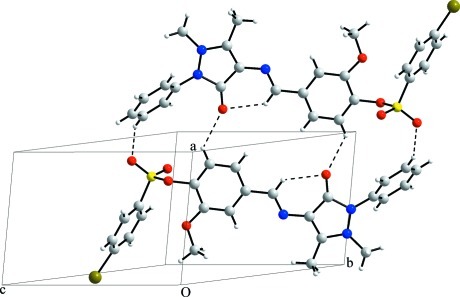
A packing diagram of the title compound, with hydrogen bonds drawn as dashed lines.

## supplementary crystallographic information

### Comment

There has been steady growth of interest in the synthesis, structure, and reactivity of Schiff bases due to their potentially biological activities such as protein and enzyme mimics (Santos *et al.*, 2001). Among the large number of compounds, 4-amino-1,5-dimethyl-2-phenylpyrazol-3-one forms a variety of Schiff bases with aldehydes, and the synthesis and crystal structures of some of them, such as (*E*)-5-[(1,5-dimethyl-3-oxo-2-phenyl-2,3-dihydro-1*H*-pyrazol-4-ylimino)methyl]-2-methoxyphenyl 4-bromobenzenesulfonate (Guo *et al.*, 2010) and (*E*)-4-[(1,5-dimethyl-3-oxo-2-phenyl-2,3-dihydro-1*H*-pyrazol-4-ylimino)methyl]phenyl 4-bromobenzenesulfonate (Han *et al.*, 2008) have been reported.

Structural information is useful when investigating the coordination properties of Schiff bases functioning as ligands. We report here the synthesis and molecular structure of the title Schiff base compound, (I), (Fig. 1)

In the title molecule (Fig. 1), bond lengths are within normal ranges (Allen *et al.*, 1987). The pyrazolone ring (C15–C17/N1/N2) is almost planar with an *r.m.s.* deviation for fitted atoms of 0.058 (2) Å. It makes a dihedral angle of 57.75 (11)° with the attached phenyl ring (C20–C25). The central benzene ring (C7–C12) makes dihedral angles of 4.41 (10), 67.09 (9) and 62.05 (10)°, respectively, with the pyrazolone ring (C15–C17/N1/N2), the bromobenzene ring (C1–C6) and the terminal phenyl ring (C20—C25).

An intramolecular C13—H13···O5═C16 hydrogen bond is found in (I) (Table 1), which helps to stabilize the conformation of the molecule. Packing is stabilized by weak, non-classical intermolecular C12—H12···O5═C16 and C22—H22···O1═S1 hydrogen bonds that form inversion related dimers (Table 1 and Fig. 2).

### Experimental

An anhydrous ethanol solution (50 ml) of 4-formyl-2-methoxyphenyl 4-bromobenzenesulfonate (3.71 g, 10 mmol) was added to an anhydrous ethanol solution (50 ml) of 4-amino-1,5-dimethyl-2-phenylpyrazol-3-one (2.03 g, 10 mmol) and the mixture stirred at 350 K for 3 h under N_2_, giving a yellow precipitate. The product was isolated, recrystallized from acetonitrile, and then dried in a vacuum to give pure compound (I) in 83% yield. Yellow single crystals of (I) suitable for X-ray analysis were obtained by slow evaporation of an acetonitrile solution.

### Refinement

The H atoms were included in calculated positions and refined using a riding model approximation. Constrained C—H bond lengths and isotropic *U* parameters: 0.93 Å and *U*_iso_(H) = 1.2*U*_eq_(C) for C*sp*^2^—H; 0.96 Å and *U*_iso_(H) = 1.5*U*_eq_(C) for methyl C—H.

### Crystal data

**Table:**

C_25_H_22_BrN_3_O_5_S	*Z* = 2
*M**_r_* = 556.43	*F*(000) = 568
Triclinic, *P*1	*D*_x_ = 1.539 Mg m^−^^3^
Hall symbol: -P 1	Mo *K*α radiation, λ = 0.71073 Å
*a* = 7.271 (2) Å	Cell parameters from 4454 reflections
*b* = 12.654 (4) Å	θ = 1.5–27.9°
*c* = 13.645 (5) Å	µ = 1.84 mm^−^^1^
α = 88.252 (15)°	*T* = 294 K
β = 85.695 (14)°	Block, yellow
γ = 73.623 (12)°	0.25 × 0.20 × 0.13 mm
*V* = 1201.0 (7) Å^3^	

### Data collection

**Table:**

Bruker SMART APEX CCD area-detector diffractometer	4225 independent reflections
Radiation source: fine-focus sealed tube	3239 reflections with *I* > 2σ(*I*)
Graphite monochromator	*R*_int_ = 0.034
φ and ω scans	θ_max_ = 25.0°, θ_min_ = 1.5°
Absorption correction: multi-scan (*SADABS*; Sheldrick, 1996)	*h* = −8→8
*T*_min_ = 0.628, *T*_max_ = 0.787	*k* = −15→15
10073 measured reflections	*l* = −16→16

### Refinement

**Table:**

Refinement on *F*^2^	Primary atom site location: structure-invariant direct methods
Least-squares matrix: full	Secondary atom site location: difference Fourier map
*R*[*F*^2^ > 2σ(*F*^2^)] = 0.025	Hydrogen site location: inferred from neighbouring sites
*wR*(*F*^2^) = 0.059	H-atom parameters constrained
*S* = 0.95	*w* = 1/[σ^2^(*F*_o_^2^) + (0.0229*P*)^2^] where *P* = (*F*_o_^2^ + 2*F*_c_^2^)/3
4225 reflections	(Δ/σ)_max_ = 0.001
319 parameters	Δρ_max_ = 0.26 e Å^−^^3^
0 restraints	Δρ_min_ = −0.40 e Å^−^^3^

### Special details

**Table:**

Geometry. All e.s.d.'s (except the e.s.d. in the dihedral angle between two l.s. planes) are estimated using the full covariance matrix. The cell e.s.d.'s are taken into account individually in the estimation of e.s.d.'s in distances, angles and torsion angles; correlations between e.s.d.'s in cell parameters are only used when they are defined by crystal symmetry. An approximate (isotropic) treatment of cell e.s.d.'s is used for estimating e.s.d.'s involving l.s. planes.
Refinement. Refinement of *F*^2^ against ALL reflections. The weighted *R*-factor *wR* and goodness of fit *S* are based on *F*^2^, conventional *R*-factors *R* are based on *F*, with *F* set to zero for negative *F*^2^. The threshold expression of *F*^2^ > 2σ(*F*^2^) is used only for calculating *R*-factors(gt) *etc*. and is not relevant to the choice of reflections for refinement. *R*-factors based on *F*^2^ are statistically about twice as large as those based on *F*, and *R*- factors based on ALL data will be even larger.

### Fractional atomic coordinates and isotropic or equivalent isotropic displacement parameters (Å^2^)

**Table:**

	*x*	*y*	*z*	*U*_iso_*/*U*_eq_	
Br1	0.01790 (3)	0.265549 (18)	0.722422 (16)	0.02781 (8)	
S1	0.77530 (8)	0.26428 (4)	0.45611 (4)	0.01811 (13)	
N1	0.4302 (2)	0.65367 (13)	0.05220 (11)	0.0157 (4)	
N2	0.2503 (2)	0.88912 (13)	−0.10094 (12)	0.0167 (4)	
N3	0.4400 (2)	0.85402 (13)	−0.14262 (12)	0.0171 (4)	
O1	0.9083 (2)	0.16360 (12)	0.48414 (10)	0.0264 (4)	
O2	0.8183 (2)	0.36677 (11)	0.46382 (10)	0.0232 (4)	
O3	0.7449 (2)	0.24482 (10)	0.34352 (9)	0.0170 (3)	
O4	0.3891 (2)	0.37409 (11)	0.33591 (10)	0.0199 (3)	
O5	0.7202 (2)	0.72221 (11)	−0.10465 (10)	0.0196 (3)	
C1	0.4676 (3)	0.36032 (16)	0.57928 (14)	0.0192 (5)	
H1	0.5192	0.4197	0.5801	0.023*	
C2	0.3033 (3)	0.36003 (17)	0.63858 (15)	0.0217 (5)	
H2	0.2443	0.4187	0.6800	0.026*	
C3	0.2296 (3)	0.27100 (17)	0.63476 (14)	0.0179 (5)	
C4	0.3105 (3)	0.18384 (16)	0.57180 (14)	0.0197 (5)	
H4	0.2549	0.1263	0.5684	0.024*	
C5	0.4742 (3)	0.18364 (16)	0.51432 (14)	0.0180 (5)	
H5	0.5318	0.1252	0.4724	0.022*	
C6	0.5533 (3)	0.27149 (16)	0.51911 (13)	0.0149 (5)	
C7	0.7089 (3)	0.33446 (15)	0.27618 (13)	0.0153 (5)	
C8	0.5194 (3)	0.40007 (16)	0.27035 (14)	0.0158 (5)	
C9	0.4833 (3)	0.48370 (15)	0.20023 (14)	0.0153 (5)	
H9	0.3591	0.5293	0.1957	0.018*	
C10	0.6326 (3)	0.49966 (15)	0.13639 (13)	0.0151 (5)	
C11	0.8185 (3)	0.43252 (15)	0.14330 (14)	0.0169 (5)	
H11	0.9176	0.4434	0.1007	0.020*	
C12	0.8570 (3)	0.34868 (16)	0.21394 (14)	0.0155 (5)	
H12	0.9812	0.3032	0.2187	0.019*	
C13	0.5975 (3)	0.58684 (16)	0.06028 (14)	0.0171 (5)	
H13	0.6991	0.5935	0.0172	0.021*	
C14	0.1955 (3)	0.44338 (17)	0.33966 (16)	0.0255 (5)	
H14A	0.1930	0.5162	0.3586	0.038*	
H14B	0.1177	0.4141	0.3868	0.038*	
H14C	0.1461	0.4463	0.2760	0.038*	
C15	0.4041 (3)	0.73576 (16)	−0.02068 (14)	0.0147 (5)	
C16	0.5449 (3)	0.76256 (15)	−0.09004 (14)	0.0155 (5)	
C17	0.2292 (3)	0.80994 (16)	−0.03330 (13)	0.0157 (5)	
C18	0.0376 (3)	0.81078 (17)	0.01264 (15)	0.0215 (5)	
H18A	−0.0359	0.7898	−0.0350	0.032*	
H18B	−0.0281	0.8834	0.0359	0.032*	
H18C	0.0528	0.7596	0.0669	0.032*	
C19	0.1022 (3)	0.94218 (16)	−0.16870 (14)	0.0205 (5)	
H19A	0.1055	0.8928	−0.2213	0.031*	
H19B	0.1265	1.0085	−0.1952	0.031*	
H19C	−0.0221	0.9598	−0.1338	0.031*	
C20	0.5247 (3)	0.93260 (16)	−0.19093 (14)	0.0158 (5)	
C21	0.6437 (3)	0.89948 (16)	−0.27480 (14)	0.0185 (5)	
H21	0.6640	0.8291	−0.2994	0.022*	
C22	0.7329 (3)	0.97310 (17)	−0.32194 (15)	0.0224 (5)	
H22	0.8145	0.9516	−0.3780	0.027*	
C23	0.7003 (3)	1.07778 (17)	−0.28548 (15)	0.0237 (5)	
H23	0.7597	1.1267	−0.3173	0.028*	
C24	0.5799 (3)	1.11040 (17)	−0.20180 (16)	0.0250 (5)	
H24	0.5580	1.1812	−0.1779	0.030*	
C25	0.4917 (3)	1.03730 (16)	−0.15343 (15)	0.0213 (5)	
H25	0.4118	1.0584	−0.0968	0.026*	

### Atomic displacement parameters (Å^2^)

**Table:**

	*U*^11^	*U*^22^	*U*^33^	*U*^12^	*U*^13^	*U*^23^
Br1	0.02550 (15)	0.03136 (14)	0.02661 (14)	−0.01081 (11)	0.00783 (10)	0.00295 (10)
S1	0.0170 (3)	0.0235 (3)	0.0133 (3)	−0.0052 (3)	−0.0002 (2)	0.0012 (2)
N1	0.0179 (11)	0.0164 (9)	0.0142 (9)	−0.0067 (8)	−0.0029 (8)	0.0001 (7)
N2	0.0134 (10)	0.0186 (9)	0.0175 (9)	−0.0038 (8)	0.0007 (8)	0.0001 (7)
N3	0.0138 (10)	0.0156 (9)	0.0207 (9)	−0.0029 (8)	0.0010 (8)	0.0033 (7)
O1	0.0178 (9)	0.0331 (9)	0.0210 (8)	0.0042 (8)	−0.0011 (7)	0.0077 (7)
O2	0.0263 (9)	0.0306 (9)	0.0179 (8)	−0.0170 (7)	0.0012 (7)	−0.0030 (7)
O3	0.0241 (9)	0.0141 (7)	0.0113 (7)	−0.0032 (7)	0.0004 (6)	0.0013 (6)
O4	0.0146 (8)	0.0236 (8)	0.0197 (8)	−0.0038 (7)	0.0027 (6)	0.0060 (6)
O5	0.0126 (9)	0.0197 (8)	0.0254 (8)	−0.0033 (7)	−0.0017 (7)	0.0050 (6)
C1	0.0214 (13)	0.0184 (11)	0.0202 (11)	−0.0095 (10)	0.0006 (10)	−0.0022 (9)
C2	0.0236 (14)	0.0204 (12)	0.0199 (11)	−0.0049 (11)	0.0036 (10)	−0.0058 (9)
C3	0.0157 (12)	0.0235 (12)	0.0139 (11)	−0.0055 (10)	−0.0005 (9)	0.0054 (9)
C4	0.0235 (13)	0.0158 (11)	0.0220 (12)	−0.0082 (10)	−0.0038 (10)	−0.0004 (9)
C5	0.0204 (13)	0.0163 (11)	0.0170 (11)	−0.0041 (10)	−0.0016 (9)	−0.0028 (9)
C6	0.0144 (12)	0.0184 (11)	0.0110 (10)	−0.0033 (9)	−0.0013 (9)	0.0024 (8)
C7	0.0214 (13)	0.0127 (10)	0.0115 (11)	−0.0042 (10)	−0.0024 (9)	0.0008 (8)
C8	0.0172 (13)	0.0179 (11)	0.0136 (11)	−0.0074 (10)	0.0012 (9)	−0.0031 (9)
C9	0.0154 (12)	0.0151 (11)	0.0145 (10)	−0.0018 (9)	−0.0027 (9)	−0.0024 (8)
C10	0.0191 (13)	0.0143 (11)	0.0130 (11)	−0.0064 (10)	−0.0017 (9)	−0.0028 (8)
C11	0.0180 (13)	0.0201 (11)	0.0144 (11)	−0.0092 (10)	0.0030 (9)	−0.0021 (9)
C12	0.0141 (12)	0.0152 (11)	0.0154 (11)	−0.0007 (9)	−0.0013 (9)	−0.0043 (9)
C13	0.0218 (13)	0.0178 (11)	0.0145 (11)	−0.0103 (10)	0.0002 (9)	−0.0015 (9)
C14	0.0161 (13)	0.0300 (13)	0.0303 (13)	−0.0078 (11)	0.0033 (10)	0.0007 (10)
C15	0.0159 (12)	0.0142 (10)	0.0145 (11)	−0.0052 (9)	−0.0003 (9)	−0.0023 (8)
C16	0.0196 (13)	0.0132 (11)	0.0147 (11)	−0.0054 (10)	−0.0051 (9)	0.0002 (8)
C17	0.0192 (13)	0.0181 (11)	0.0106 (10)	−0.0068 (10)	0.0001 (9)	−0.0036 (9)
C18	0.0190 (13)	0.0255 (12)	0.0190 (11)	−0.0056 (10)	0.0028 (10)	−0.0023 (9)
C19	0.0191 (13)	0.0190 (11)	0.0208 (12)	−0.0006 (10)	−0.0032 (10)	−0.0018 (9)
C20	0.0152 (12)	0.0157 (11)	0.0173 (11)	−0.0051 (9)	−0.0046 (9)	0.0045 (9)
C21	0.0196 (13)	0.0156 (11)	0.0190 (11)	−0.0021 (10)	−0.0039 (10)	0.0005 (9)
C22	0.0187 (13)	0.0296 (13)	0.0184 (12)	−0.0067 (11)	−0.0023 (10)	0.0068 (10)
C23	0.0235 (14)	0.0255 (13)	0.0267 (13)	−0.0133 (11)	−0.0113 (11)	0.0117 (10)
C24	0.0335 (15)	0.0163 (12)	0.0276 (13)	−0.0084 (11)	−0.0120 (11)	0.0019 (10)
C25	0.0247 (14)	0.0195 (12)	0.0183 (11)	−0.0033 (10)	−0.0043 (10)	−0.0007 (9)

### Geometric parameters (Å, º)

**Table:**

Br1—C3	1.893 (2)	C9—H9	0.9300
S1—O2	1.4255 (15)	C10—C11	1.387 (3)
S1—O1	1.4266 (14)	C10—C13	1.474 (3)
S1—O3	1.6045 (15)	C11—C12	1.394 (3)
S1—C6	1.751 (2)	C11—H11	0.9300
N1—C13	1.282 (2)	C12—H12	0.9300
N1—C15	1.399 (2)	C13—H13	0.9300
N2—C17	1.374 (2)	C14—H14A	0.9600
N2—N3	1.406 (2)	C14—H14B	0.9600
N2—C19	1.473 (2)	C14—H14C	0.9600
N3—C16	1.403 (2)	C15—C17	1.370 (3)
N3—C20	1.431 (2)	C15—C16	1.446 (3)
O3—C7	1.416 (2)	C17—C18	1.481 (3)
O4—C8	1.355 (2)	C18—H18A	0.9600
O4—C14	1.431 (2)	C18—H18B	0.9600
O5—C16	1.235 (2)	C18—H18C	0.9600
C1—C6	1.382 (3)	C19—H19A	0.9600
C1—C2	1.393 (3)	C19—H19B	0.9600
C1—H1	0.9300	C19—H19C	0.9600
C2—C3	1.381 (3)	C20—C21	1.382 (3)
C2—H2	0.9300	C20—C25	1.385 (3)
C3—C4	1.384 (3)	C21—C22	1.394 (3)
C4—C5	1.375 (3)	C21—H21	0.9300
C4—H4	0.9300	C22—C23	1.380 (3)
C5—C6	1.394 (3)	C22—H22	0.9300
C5—H5	0.9300	C23—C24	1.384 (3)
C7—C12	1.369 (3)	C23—H23	0.9300
C7—C8	1.401 (3)	C24—C25	1.392 (3)
C8—C9	1.387 (3)	C24—H24	0.9300
C9—C10	1.396 (3)	C25—H25	0.9300
			
O2—S1—O1	120.80 (10)	C11—C12—H12	120.5
O2—S1—O3	109.18 (8)	N1—C13—C10	121.44 (19)
O1—S1—O3	103.54 (8)	N1—C13—H13	119.3
O2—S1—C6	109.56 (9)	C10—C13—H13	119.3
O1—S1—C6	107.69 (9)	O4—C14—H14A	109.5
O3—S1—C6	104.84 (9)	O4—C14—H14B	109.5
C13—N1—C15	119.41 (18)	H14A—C14—H14B	109.5
C17—N2—N3	106.70 (15)	O4—C14—H14C	109.5
C17—N2—C19	123.01 (18)	H14A—C14—H14C	109.5
N3—N2—C19	116.10 (15)	H14B—C14—H14C	109.5
C16—N3—N2	109.69 (16)	C17—C15—N1	122.44 (19)
C16—N3—C20	123.24 (17)	C17—C15—C16	108.33 (17)
N2—N3—C20	119.48 (16)	N1—C15—C16	129.17 (18)
C7—O3—S1	120.03 (12)	O5—C16—N3	123.13 (18)
C8—O4—C14	118.06 (15)	O5—C16—C15	132.29 (18)
C6—C1—C2	119.3 (2)	N3—C16—C15	104.55 (17)
C6—C1—H1	120.3	C15—C17—N2	109.88 (19)
C2—C1—H1	120.3	C15—C17—C18	128.98 (19)
C3—C2—C1	118.8 (2)	N2—C17—C18	121.13 (18)
C3—C2—H2	120.6	C17—C18—H18A	109.5
C1—C2—H2	120.6	C17—C18—H18B	109.5
C2—C3—C4	122.1 (2)	H18A—C18—H18B	109.5
C2—C3—Br1	118.73 (16)	C17—C18—H18C	109.5
C4—C3—Br1	119.14 (16)	H18A—C18—H18C	109.5
C5—C4—C3	118.9 (2)	H18B—C18—H18C	109.5
C5—C4—H4	120.5	N2—C19—H19A	109.5
C3—C4—H4	120.5	N2—C19—H19B	109.5
C4—C5—C6	119.7 (2)	H19A—C19—H19B	109.5
C4—C5—H5	120.2	N2—C19—H19C	109.5
C6—C5—H5	120.2	H19A—C19—H19C	109.5
C1—C6—C5	121.1 (2)	H19B—C19—H19C	109.5
C1—C6—S1	119.14 (16)	C21—C20—C25	121.3 (2)
C5—C6—S1	119.56 (16)	C21—C20—N3	117.67 (18)
C12—C7—C8	122.31 (17)	C25—C20—N3	120.96 (19)
C12—C7—O3	119.05 (17)	C20—C21—C22	119.0 (2)
C8—C7—O3	118.43 (18)	C20—C21—H21	120.5
O4—C8—C9	126.79 (18)	C22—C21—H21	120.5
O4—C8—C7	115.05 (16)	C23—C22—C21	120.1 (2)
C9—C8—C7	118.16 (19)	C23—C22—H22	120.0
C8—C9—C10	120.31 (19)	C21—C22—H22	120.0
C8—C9—H9	119.8	C22—C23—C24	120.4 (2)
C10—C9—H9	119.8	C22—C23—H23	119.8
C11—C10—C9	120.14 (18)	C24—C23—H23	119.8
C11—C10—C13	118.52 (19)	C23—C24—C25	120.0 (2)
C9—C10—C13	121.34 (18)	C23—C24—H24	120.0
C10—C11—C12	120.16 (19)	C25—C24—H24	120.0
C10—C11—H11	119.9	C20—C25—C24	119.0 (2)
C12—C11—H11	119.9	C20—C25—H25	120.5
C7—C12—C11	118.90 (19)	C24—C25—H25	120.5
C7—C12—H12	120.5		

### Hydrogen-bond geometry (Å, º)

**Table:**

*D*—H···*A*	*D*—H	H···*A*	*D*···*A*	*D*—H···*A*
C13—H13···O5	0.93	2.32	3.020 (3)	132
C22—H22···O1^i^	0.93	2.51	3.394 (3)	158
C12—H12···O5^i^	0.93	2.52	3.212 (3)	131

Symmetry code: (i) −*x*+2, −*y*+1, −*z*.
